# Supplementary material for: Are carbon emissions trading and green financial instruments synergistic? -Comprehensive quantitative research based on content analysis
Source: PLoS One. 2024 Mar 7;19(3):e0298601. doi: 10.1371/journal.pone.0298601 (PMC10919601; doi:10.1371/journal.pone.0298601)
Supplement: S1 Data — (ZIP) [file pone.0298601.s001.zip › Data/地级市Z_F工作报告.zip!/╕╩╦α/╠∞╦«╩╨/└┤╫╘╣1⁄2╓┌║┼í╛┬φ┐╦╩2╛▌═°í┐_2012.docx]

各位代表:

现在,我代表市人民政府,向大会报告过去五年的工作,提出今后五年政府工作的奋斗目标和主要任务,请予审议,并请各位政协委员和列席人员提出意见、建议。

过去五年政府工作回顾

市五届人民代表大会第一次会议以来,在市委的坚强领导下,我们紧紧依靠全市人民,全面贯彻落实科学发展观,抢抓国家西部大开发、扩大内需等良好机遇,努力克服国际金融危机和“5·12”地震等诸多不利影响,全力实施《关中—天水经济区发展规划》和省委、省政府“中心带动、两翼齐飞、组团发展、整体推进”及我市“1135”区域发展战略,开拓创新,真抓实干,圆满完成了各项目标任务,经济社会步入了科学发展、快速发展、和谐发展的新阶段。预计2011年,全市实现生产总值349亿元,比2006年增长71.2%,年均增长11.4%。其中,第一产业增加值68亿元,年均增长9.2%;第二产业增加值136亿元,年均增长12.3%;第三产业增加值145亿元,年均增长11.4%。大口径财政收入60亿元,是2006年的3.23倍,年均增长26.39%;财政支出145亿元,是2006年的3.7倍,年均增长29.9%。社会消费品零售总额150亿元,比2006年增长1.35倍,年均增长18.6%。接待游客830万人次,创旅游综合收入45亿元,分别比2006年增长3倍和5.1倍。城镇居民人均可支配收入13002元,比2006年净增5417元,年均增长11.4%;农民人均纯收入3248元,比2006年净增1583元,年均增长14.3%。

五年来,我们群策群力,抓项目促发展,抓改革促开放,抓民生促和谐,各项工作都取得了新进展。

坚持实施项目带动战略,投资拉动效应明显增强。我们把项目建设作为加快发展的重要支撑,精心谋划,增加储备,五年累计完成全社会固定资产投资987.1亿元,年均增长31.2%。共组织实施重大项目217个;分四批争取到国家扩大内需项目397个,总投资51.91亿元,累计完成投资39.3亿元;1286个地震灾后恢复重建项目完成投资71.04亿元,全面完成了114个整村搬迁等重建任务,城乡居民住房、交通道路和教育、卫生基础设施得到改善。积极组团参加国内外重大经贸洽谈活动,全方位推介项目,广泛开展招商引资,五年累计签约招商引资项目919项,到位资金204.5亿元。天水军民合用机场开通民航,宝天、天定高速公路建成通车,宝天高速天水过境段即将竣工,天平铁路、庄天二级公路、天然气管道中卫至贵阳联络线等工程加快建设。城乡电网投资9.04亿元,新改建高、中、低压变电站台275处、线路1000.2公里。大唐甘谷电厂、中材水泥等一批产业项目建成投产,28个重点节能和循环经济项目累计完成投资8.9亿元。这些重大基础设施、产业和民生项目的实施,缓解了长期以来制约我市发展的交通、能源等瓶颈,优化了发展环境和经济结构,有力地拉动了经济社会的快速发展。

坚持实施工业强市战略,工业转型升级步伐加快。工业园区加快建设,集聚效应日益显现,天水经济技术开发区晋升为国家级开发区。星火机床、华天电子、长城电工电器三大产业园启动建设,县区工业园发展势头良好,“一区多园”格局初步形成。坚持“引大引强”,主动加强与大型企业集团、科研院所的战略合作,总投资164.65亿元的9个央企合作工业项目成功签约。五年共实施工业项目260多个,总投资237亿元,累计完成投资136.8亿元。坚持扶优扶强,年销售收入过亿元的企业达到22户,其中10亿元以上企业4户,华天科技上市,星火公司跨国并购重组,机械制造、电工电器、电子信息、建筑材料、医药食品、能源化工等六大产业不断壮大。污染减排各项约束性指标全面完成。企业技术创新能力不断增强,研发新产品300多项,建成国家级企业技术中心4个、省级11个,天水长城电器试验研究所晋升为国家高低压电器质量监督检验中心,我市被认定为国家先进制造高新技术产业化基地;荣获中国名牌产品1个,中国驰名商标9个,成功跻身国家商标战略实施示范城市行列。

坚持实施农业稳市战略,农业和农村经济快速发展。深入实施科技增粮工程,粮食生产连续五年取得较好收成。天水农业高新技术示范区晋升为国家级农业科技园区,设施农业、旱作农业等新技术全面推广,农产品地理标志保护工作进一步加强,我市成为全国首批循环农业示范市。“一城两园一集团”启动建设,新建城郊南山十万亩花牛苹果、渭河河谷川道设施蔬菜、张家川和清水肉牛养殖等一批产业基地,果品、蔬菜、畜牧三大主导产业和马铃薯、中药材、花卉等特色产业不断壮大。2011年,全市干鲜果基地面积315万亩,蔬菜种植面积105.5万亩,畜禽饲养量1906.6万头(只),分别比2006年增长102.5%、104.6%、92.8%。现有市级以上重点龙头企业83家、农民专业合作社477家。新农村建设“百村示范、千村推进”工程和城乡一体化试点稳步推进,8个省级试点村和101个市级示范村基本实现了“八通六有”,29个乡镇列入省、市级小城镇综合改革试点。农业生产条件持续改善,累计完成生态造林144.9万亩,兴修梯田97.6万亩,治理水土流失1177.4平方公里,新增节水灌溉面积11万亩,建成户用沼气10.7万户,解决了102.27万人的安全饮水问题。

坚持实施商贸活市战略,城乡市场繁荣活跃。全面贯彻落实扩大内需政策,努力开拓城乡市场,电子商务、连锁经营、物流配送、农超对接等新型业态快速发展,市场体系不断完善。苏宁电器、国美电器和宝商连锁超市等知名企业落户我市,麦积桥南家居建材城、秦州七里墩建材市场和天宝家居装饰建材城等专业市场建成运营。麦积甘泉现代物流园区和陇东南区域粮食物流中心加快建设,“万村千乡”等市场建设工程扎实推进,新建、改建各类市场120处。加强“菜篮子”工程建设,采取“农超对接”、政府储备和财政补贴等措施,有效保障了市场供给。对外贸易大幅增长,进出口总额由2006年的9001万美元预计增加到2011年的2.7亿美元。坚持举办“金融活动周”,交通银行、兰州银行入驻天水,金融对经济社会发展的支持力度不断加大。预计2011年底,金融机构本外币各项存贷款余额分别比2006年底增长1.73倍和2.57倍。

坚持实施旅游富市战略,文化旅游业加快发展。五年共组织实施1000万元以上旅游项目48个,累计完成投资15.8亿元。伏羲庙、胡氏民居、大像山、水帘洞等景区景点建设和保护维修项目基本完成,世行贷款麦积山保护与开发、清水温泉生态游乐园、麦积区农家乐改造提升工程等项目加快建设,兰州饭店天水和谐园等一批星级宾馆饭店建成运营,麦积山风景名胜区晋升为国家5A级旅游景区,玉泉观、南郭寺、伏羲庙成功晋级国家4A级旅游景区,以“农家乐”为主的乡村旅游业蓬勃发展,旅游基础条件得到改善,服务接待能力明显提升,我市在第三届博鳌国际旅游论坛上被评为国家精品旅游城市。大力培育文化旅游品牌,积极推进历史文化与旅游产业的深度融合,启动了全球华人寻根祭祖圣地和伏羲文化、大地湾文化、麦积山石窟艺术展示基地建设前期工作,先后涌现出《伏羲颂》、《麦积圣歌》、《山里红》、《邓宝珊将军》、《关山月》等精品剧目,“陇右讲堂”、“相聚天水合唱节”等特色文化品牌知名度日益提升,飞天雕漆、汉唐陶艺、鸳鸯玉器、大漠麻鞋、庞公玉器等特色旅游产品走俏市场。

坚持实施科教兴市战略,社会事业全面进步。创新型城市建设步伐不断加快,连续保持了“全国科技进步先进市”称号。人才工作机制不断完善,人才培养和引进工作全面加强。成立了旅游和装备制造业两个职业教育集团,职业教育服务主导产业和助力经济发展的作用初步显现。以县为主的农村义务教育管理体制和经费保障机制不断完善,“两基”成果得到巩固提高,各级各类教育健康发展,教师队伍建设进一步加强,教育教学质量不断提升。五年累计投入教育99.5亿元,是上一个五年的3倍,中小学校舍安全、儿童美德教育和特殊教育学校建设等工程加快实施,新建、改建中小学校舍123.6万平方米,教育布局得到优化,办学条件明显改善。医药卫生体制改革深入推进,公共卫生工作全面加强,医疗卫生服务体系和基础设施进一步完善,我市荣获全省卫生城市“五连冠”。文物和非物质文化遗产保护工作全面加强,博物馆、文化馆、图书馆和乡镇综合文化站、农家书屋等文化基础设施条件不断改善,城乡节日文化活动丰富多彩。健身器材进村入户,群体活动广泛开展,竞技体育水平不断提升。广播电视电影事业快速发展。人口与计划生育工作目标全面实现。第六次全国人口普查工作顺利完成。第二轮修志工作进展顺利。市残疾人综合服务中心基本建成。城镇社区建设得到加强。国土资源、工商管理、普法教育、外事侨务、地方志、统计、人防、气象、防震等工作取得新进展。妇女儿童、老龄、老干部和残疾人权益得到保护。国防动员、双拥共建和精神文明建设成效显著,我市荣获全国双拥模范城市“三连冠”和全省文明城市标兵称号。
